# Effects of solid lipid nanocarrier containing methyl urolithin A by coating folate-bound chitosan and evaluation of its anti-cancer activity

**DOI:** 10.1186/s12896-024-00845-6

**Published:** 2024-04-10

**Authors:** Ilham Naeem Abd Ali Al-Fatlawi, Vahid Pouresmaeil, Fatemeh Davoodi-Dehaghani, Aida Pouresmaeil, Ali Akhtari, Masoud Homayouni Tabrizi

**Affiliations:** 1 Department of Biology, Science and Research Branch, Islamic Azad University, Tehran, Iran; 2 Department of Biochemistry, Faculty of Medicine, Mashhad Medical Sciences, Islamic Azad University, Mashhad, Iran; 3 Department of Biology, Faculty of Basic Sciences, Central Tehran Branch, Islamic Azad University, Tehran, Iran; 4 Department of Biology, Mashhad Branch, Islamic Azad University, Mashhad, Iran

**Keywords:** Methyl urolithin-A, Apoptosis, Solid Lipid Nanoparticles, Chitosan, Folate, Cancer,Antioxidant

## Abstract

**Background:**

Nanotechnology-based drug delivery systems have received much attention over the past decade. In the present study, we synthesized Methyl Urolithin A-loaded solid lipid nanoparticles decorated with the folic acid-linked chitosan layer called MuSCF-NPs and investigated their effects on cancer cells.

**Methods:**

MuSCF-NPs were prepared using a high-pressure homogenization method and characterized using FTIR, FESEM, DLS, and zeta potential methods. Drug encapsulation was assessed by spectrophotometry and its cytotoxic effect on various cancer cells (MDA-*MB231*, MCF-7, PANC, AGS, and HepG2) by the MTT method. Antioxidant activity was assessed by the ABTS and DPPH methods, followed by expression of genes involved in oxidative stress and apoptosis by qPCR and flow cytometry.

**Results:**

The results showed the formation of monodisperse and stable round nanoparticles with a size of 84.8 nm. The drug loading efficiency in MuSCF-NPs was reported to be 88.6%. MuSCF-NPs exhibited selective cytotoxicity against MDA-*MB231* cells (IC_50_ = 40 μg/mL). Molecular analysis showed a significant increase in the expression of Caspases 3, 8, and 9, indicating that apoptosis was occurring in the treated cells. Moreover, flow cytometry results showed that the treated cells were arrested in his SubG1 phase, confirming the pro-apoptotic effect of the nanoparticles. The results indicate a high antioxidant effect of the nanoparticles with IC_50_ values ​​of 45 μg/mL and 1500 μg/mL against ABTS and DPPH, respectively. The reduction of catalase gene expression confirmed the pro-oxidant effect of nanoparticles in cancer cells treated at concentrations of 20 and 40 μg/mL.

**Conclusions:**

Therefore, our findings suggest that the MuSCF-NPs are suitable candidates, especially for breast cancer preclinical studies.

## Background

Breast cancer continues to be a significant cause of mortality worldwide in 2023. Despite advances in early detection and treatment, breast cancer remains the leading cause of cancer-related deaths among women globally. According to the World Health Organization (WHO), breast cancer is responsible for an estimated 685,000 deaths in 2020, and the incidence of breast cancer continues to rise. The impact of breast cancer extends beyond the individual patient and affects families, communities, and society as a whole. Breast cancer mortality rates are particularly high in low- and middle-income countries, where access to early detection and treatment is limited. This underscores the need for increased awareness, education, and resources to address breast cancer on a global scale. Efforts are underway to improve breast cancer outcomes and reduce mortality rates through research, advocacy, and policy initiatives. These efforts include expanding access to screening and diagnostic tools, developing more effective treatments, and improving support services for patients and survivors [1]. As we continue to make progress in the fight against breast cancer, it is essential to recognize the most efficient and safe anticancer compounds to improve outcomes for patients worldwide.

Research has demonstrated the anti-cancer properties of plants, microorganisms, and their metabolites like urolithins. Methyl urolithin A (MuA), a naturally occurring compound derived from specific plant sources, has garnered significant attention due to its remarkable potential in various biological activities. In recent years, researchers have been exploring novel methods to enhance the stability and bioavailability of MuA [2]. More than half of the cancer drugs used today are derived from plants, microorganisms, and marine organisms [3]. One of the main anticancer mechanisms attributed to the anticancer compounds is their potential to induce oxidative stresses in cancer cells, which are responsible for the induction of apoptotic response and cellular death. The apoptotic death mediated by oxidative storms potentially occurs by modifying the apoptotic caspase genes such as Caspase 3, 8, and 9, which are being down-regulated in cancer cells [4].

Oxidative stress (OS) has been identified as a contributing factor in cancer development. On the other hand, the optimized antioxidant defense system in cancer cells such as Catalase makes them resistant to internal-originated oxidative storms [5]. Moreover, the rapid proliferation of cancer cells and the development of colonies necessitate a continuous non-stop growth of cell-feeding pathways providing alternative complex blood vessels around the tumor mass [6]. In this regard, cancer cells severely consume input carbon sources such as folate for DNA replication and cell proliferation process, which is met by up-regulating the membrane folate receptors.

Investigating natural-based anticancer compounds targeting the cancer cell antioxidant defense response and folate receptor-mediated cellular uptake can efficiently induce a selective cytotoxic impact on cancer cells expressing large numbers of folate receptors. Folate receptors are overexpressed in various types of human cancers, including breast, colon, lung, liver, ovarian, and pancreatic cancer cells [7–10]. In this regard, considering the limited chemical stability, non-polar structure, and weak aqueous solubility of natural-originated bioactive compounds, nanotechnology has opened novel promising horizons in cancer treatment strategies by presenting the applicable nano drug delivery systems [11–14]. These systems have played a crucial role in delivering hydrophobic. In other words, encapsulating bioactive compounds into nanocarriers enhances their chemical stability by protecting them from enzymatic degradation, prolonging blood circulation interval, and improving their cell selective cellular uptake, which altogether enhances overall safety and efficiency [15].

Nanotechnology-based drug delivery systems can be classified into inorganic, polymeric, and lipid-based nanoparticles based on chemical composition and structure. Lipid-based nanoparticles have been studied over the past decades for their various advantages, including improved biocompatibility, bioavailability, drug delivery, and simple formulation.

Lipid-based nanoparticles include non-ionic surfactants, liposomes, lipid nanoparticles, and solid lipid nanoparticles (SLN). Both hydrophilic and hydrophobic drugs can be encapsulated in SLNs with greater entrapment efficiencies compared with liposomes [16]. Also, drug release from SLNs is controllable by modifying the structural lipid components [17, 18]. SLN molecules and chitosan polymers can be modified by folic acid, antibodies, integrins, transferrin, and polysaccharides to selectively be delivered to specific tissues and increase [19, 20] by targeting the individual cellular receptors.

There are several types of anticancer phytochemicals have been loaded in SLN/liposomes and coated with chitosan nanoparticles such as curcumin [21], crocin [22], thymol [23], and kaempferol [24]. Herein, methyl urolithin-A (MuA) was selected due to its natural bio-sources found in human gut microbiota and its anticancer potentials [25]. It is produced by ellagic acid (EA) metabolization conducted by human gut microbiota. EA is found in pomegranates, berries, and nuts and exhibits several bio-activities including antioxidant, anti-proliferative, anti-inflammatory, and anti-oxidant potentials [26, 27].

In the current study, MuA was selected as the cancer cell cytotoxic bioactive compound to evaluate its selective cellular uptake to the human MDA-MB-231 breast cancer cells by designing novel SLN nanoparticles decorated with folate-linked chitosan layer.

## Materials and methods

Methyl urolithin A (MUA) (Golexir Pars, Mashhad, Iran); ABTS, DPPH, DAPI staining, the Stearic acid, the lecithin, folic acid, Chitosan, Propidium Iodide (PI), Acridine Orange (AO), and MTT purchased from Sigma Aldrich (Germany). Cancer cell lines MCF-7, MDA-MB-231, HepG2, PANC, AGS, and normal HDF fibroblast cells were obtained from Pasteur Institute (Iran), and the Primers were purchased from Takapoo Zist (Iran). Cell culture materials and reagents were provided by GIBCO (USA).

### MuSCF-NPs synthesis

According to the previous study, to prepare SLN nanoparticles, a high-energy homogenization method was used applying a probe-mediated sonicate homogenizer [28]. Briefly, the lecithin was added to stearic acid at a proportion of 2:1 (W/W). Then, Tween 80 was dissolved in 1 mL of dichloromethane and added to the MuA (10 mg) solution. The resulting mixture was mixed with the solution containing the stearic acid-lecithin mixture. After loading MuA onto SLN-NPs, folic acid was linked to chitosan by applying 1-ethyl-3-(3-dimethylaminopropyl) carbodiimide hydrochloride (EDC), and N-hydroxysuccinimide (NHS) to activated carboxyl and amine group, respectively. The produced folate-linked chitosan was then decorated with the SLN nanoparticles.

### Characterization of MuSCF-NPs

The dehydrated and hydrodynamic size dimensions of the MuSCF-NPs were determined using Field emission scanning electron microscopy (FESEM) and dynamic light scattering (DLS) techniques, respectively. DLS analysis was performed at pH = 7.4 and 37 °C. The FESEM involved drying a 50-µL sample drop on aluminum foil and coating it with a thin ionic gold layer for FESEM imaging. The chemical composition of the MuSCF-NPs was characterized using FTIR analysis, where MuSCF-NPs were mixed with potassium bromide (KBr) and compressed into a thin disc for recording the FTIR spectrums. The nanoparticles’ surface charge was measured utilizing a Zetasizer device (nanoparticle SZ-100).

### Encapsulation efficiency (EE) and the percent of folate binding

The Encapsulation Efficiency (EE%) was evaluated by measuring the MuA absorbance before and after the formulation process. Briefly, an absorption spectrophotometer was used to measure the MuA absorbance at its standard concentrations. The plotted standard curve was applied to estimate the MuA concentration before and after the loading process as the following equation:

$$\mathrm{EE\% }= ({[{\text{MuA}}]}_{{\text{A}}} / {[{\text{MuA}}]}_{{\text{B}}}) \times 100$$

To determine the folate conjugation efficiency (FCE%), the folic acid standard curve was plotted by recording the folic acid absorbance at 290 nm at its provided standard concentrations (0.25, 0.5, 1, and 2 mg/mL). The FCE% was estimated considering the plotted standard curve.

### Cytotoxicity evaluation of nanoparticles

Cancerous cell lines (AGS, PANC, HepG2, MDA-MB-231, and MCF-7) and normal HDF cell lines were cultured in a complete DMEM cell culture medium under standard conditions for 24 h. The medium was supplemented with FBS (10%) and streptomycin/penicillin. The 24- cells were seeded in 96-well plates (5 × 10^3^ cells/well density) and cultured for 24 h. The cultured cells were exposed to a range of MuSCF-NPs doses (7.8, 15.6, 31.2, 62.5, 125, 250, and 500 µg/mL). The exposed cells were cultured for a further 48-h incubation at the standard conditions. Afterward, the MTT-containing medium was replaced and incubated for 3 h at 37 °C to reduce the MTT dye to formazan, which is used as the live cell index. Then, the produced formazan was dissolved in DMSO to record its absorbance at 570 nm applying a Stat fax 2100 plate reader. The cells' survival was measured by applying the following equation [29].

$$Vitality\ percentage= absorption\ of\ sample/absorption\ of\ control *100$$

### Antioxidant capacity of MuSCF-NPs

To assess the antioxidant activity of MuSCF-NPs, their ability to scavenge radicals was determined using a previously described method [20]. In brief, activated ABTS and DPPH solutions were prepared. The ABTS solution (7 mM) was activated with potassium persulfate (2.45 mM) at a 1:1 ratio, diluted with water (1:1 V/V), and stored in the dark at 25 °C for 14 h. Additionally, an ethanolic DPPH solution was prepared by dissolving 1 mg of DPPH in 17 mL of ethanol [20, 30].

$$\mathrm{ IRA\%\ or\ IRD\% = (A\ control - A\ sample) / A\ control \times 100}$$

### Catalase gene expression

Furthermore, the Mu-PFCNPs have been found to significantly decrease the activity of SOD, an enzyme involved in the antioxidant defense system (Fig. 4). Interestingly, the increased presence of Mu-PFCNPs resulted in an upregulation of SOD gene expression, suggesting that these nanoparticles possess pro-oxidant properties that work in conjunction with their cytotoxic effects. This primary down-regulation of SOD may have a positive impact on the dismutation of superoxide radicals, ultimately enhancing the potential for anticancer activity [31]. In contrast, the expression of the CAT gene was not significantly affected by the presence of Mu-PFCNPs. This suggests that these nanoparticles do not have a direct impact on the CAT-mediated antioxidant response in cells. However, this lack of impact on CAT expression may be beneficial for normal cells that rely on the CAT-mediated antioxidant defense pathway [32].

### Apoptotic study

#### Evaluation of cell cycle

Flow cytometry and PI dye were used to evaluate the cell cycle and induction of apoptosis by nanoparticles. For this purpose, cells were cultured, and treated with different concentrations of nanoparticles determined from the MTT results after 24 h. After 48 h, the medium was removed, and the cells were trypsinized from the bottom of the plate and transferred to microtubes. After centrifugation, the supernatant was drained and the cells were washed three times. PI dyes containing triton were then added to the cell sediment and analyzed by flow cytometry.

#### Evaluation of the expression level of apoptotic genes

Real-time qPCR was used to assess the expression of apoptosis-related genes (CAT, Cas-3, Cas-8, Cas-9) using specific primers whose sequences are listed in Table 1. Cells were exposed to various concentrations of her MuSCF-NPs for 48 h. After incubation, we extracted RNA from the cells, synthesized cDNA, and performed real-time qPCR.

**Table 1:** The sequence of target gene primer sets

Gene	Forward	Reverse
GAPDH	GCAGGGGGGAGCCAAAAGGGT	TGGGTGCCAGTGATGGCATGG
CAT	CGTGCTGAATGAGGAACAGA	AGTCAGGGTGGACCTCAGTG
CAS-3	CTGGACTGTGGCATTGAGAC	ACAAAGCGACTGGATGAACC
CAS-8	GAAAAGCAAACCTCGGGGATAC	CCAAGTGTGTTCCATTCCTGTC
CAS-9	CCAGAGATTCGCAAACCAGAGG	GAGCACCGACATCACCAAATCC

### Statistical analysis

Data were analyzed with GraphPad Prism (V.8.0) and significant differences were determined at *p*-value < 0.05. All experiments were repeated thrice for reproducibility and accuracy. All data were expressed as mean ± SD.

## Results

### Characterization of MuSCF-NPs

**Fig. 1:**
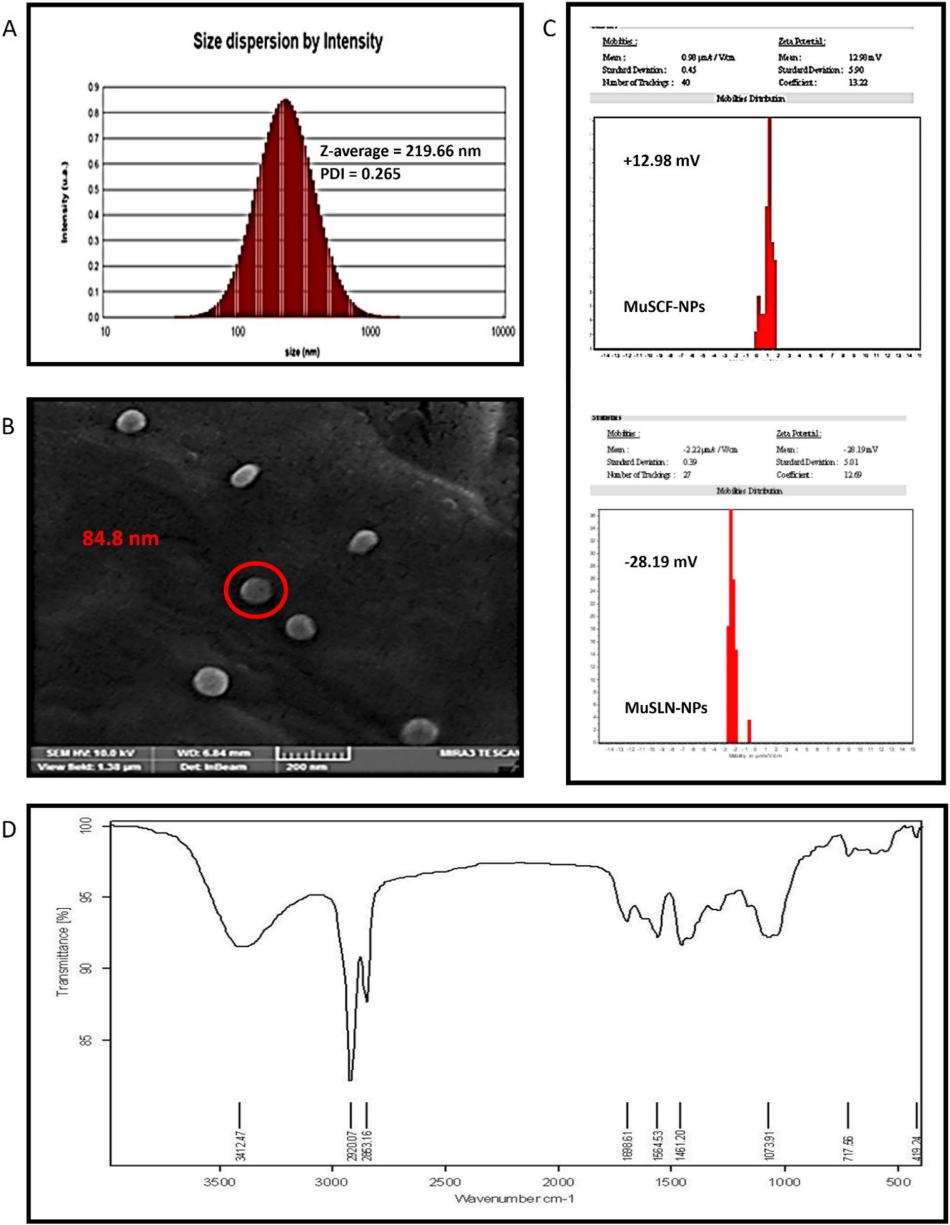
Characterization of MUA-SCF-NPs. **A** DLS reported size estimation; **B** The morphology of nanoparticles by FESEM. **C** Zeta potential (Surface charge). **D** The FTIR analysis of MuSCF-NPs. MuSLN-NPs: MuA-loaded SLN nanoparticles; MuSCF-NPs: MuA-loaded SLN-Chotosan-folate nanoparticles

The size of modified nanoparticles was evaluated using the DLS method. The results showed the formation of nanoparticles with an average diameter of 84.8 nm (uncoated nanoparticles 69.77 nm), an average hydrodynamic diameter of 219.6 nm (uncoated nanoparticles 178.5 nm), and a dispersion index of 0.26. As can be seen, the modified nanoparticles are larger than the uncoated nanoparticles, which can be attributed to the presence of chitosan-folate on the surface of the nanoparticles (Fig. 1A).

The presence of lecithin and stearic acid in the structure of nanoparticles is responsible for the negative surface charge of nanoparticles. The synthesized nanoparticles have a negative surface charge of -28 mV. A surface charge higher than ± 25 mV indicates the formation of particles with proper stability because the particles have enough force to repel and prevent agglomeration. After coating, the surface charge of nanoparticles changed from negative to positive. The charge modification is attributed to the presence of chitosan and folic acid on the surface of the nanoparticles. The particles’ surface charge was measured at $\sim$+13 mV indicating the synthesized nanoparticles' average stability (Fig. 1B).

The FESEM reported results indicated the spherical nanoparticles’ morphology and their dehydrated size at 100 nm, which is consistent with DLS results (Fig. 1C).

The FTIR spectrum for MuSCF-NPs is presented in Fig. 1D. The peaks at 2863.1 to 2920.5 cm^−1^ (O–H and C-H stretching) and 1073.9 cm^−1^ (single bonds for C–C and C-O) wavenumbers correspond to the chemical structure of triglyceride and tween-80 as the SLN compositions [33]. The peak at 1698 cm^−1^ (stretching C = O) indicates the FA’s and chitosan carboxyl group and its successful conjugation and decoration process on the SLN nanocarriers [34, 35] (Fig. 1D), Finally, the broad peak for OH and -NH groups was detected at 3412.47 cm^−1^, which confirms the existence of decorated chitosan layer on the SLN nanocarriers.

#### Encapsulation of methyl urolithin A in MuSCF-NPs

**Fig. 2:**
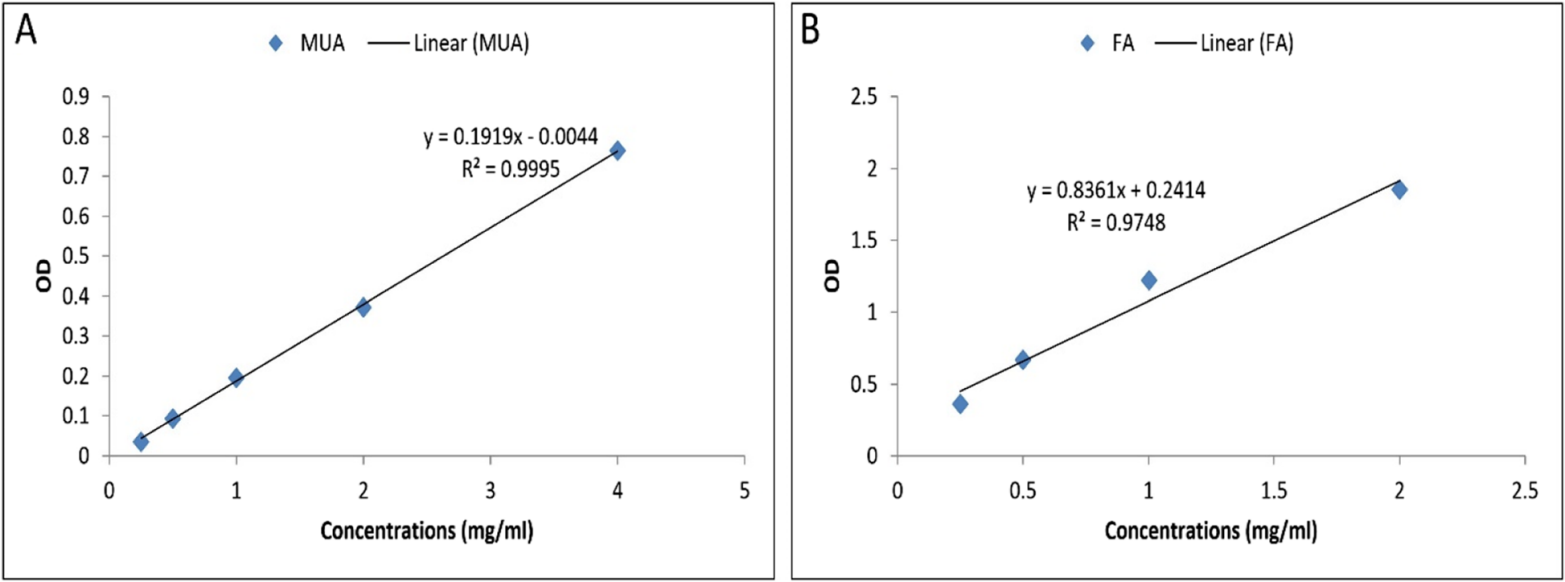
The MuA and Folate standard curves

Based on the obtained results from the standard curve, the amount of encapsulated drug was reported as 88.6%. Moreover, considering the folic acid standard curve (Fig. 2), 0.821 mg of folic acid was estimated among the total 2 mg of folate conjugating efficiency was estimated at 58.9% (Fig. 2).

### Antioxidant power of MuSCF-NPs

#### ABTS and DPPH results

**Fig. 3:**
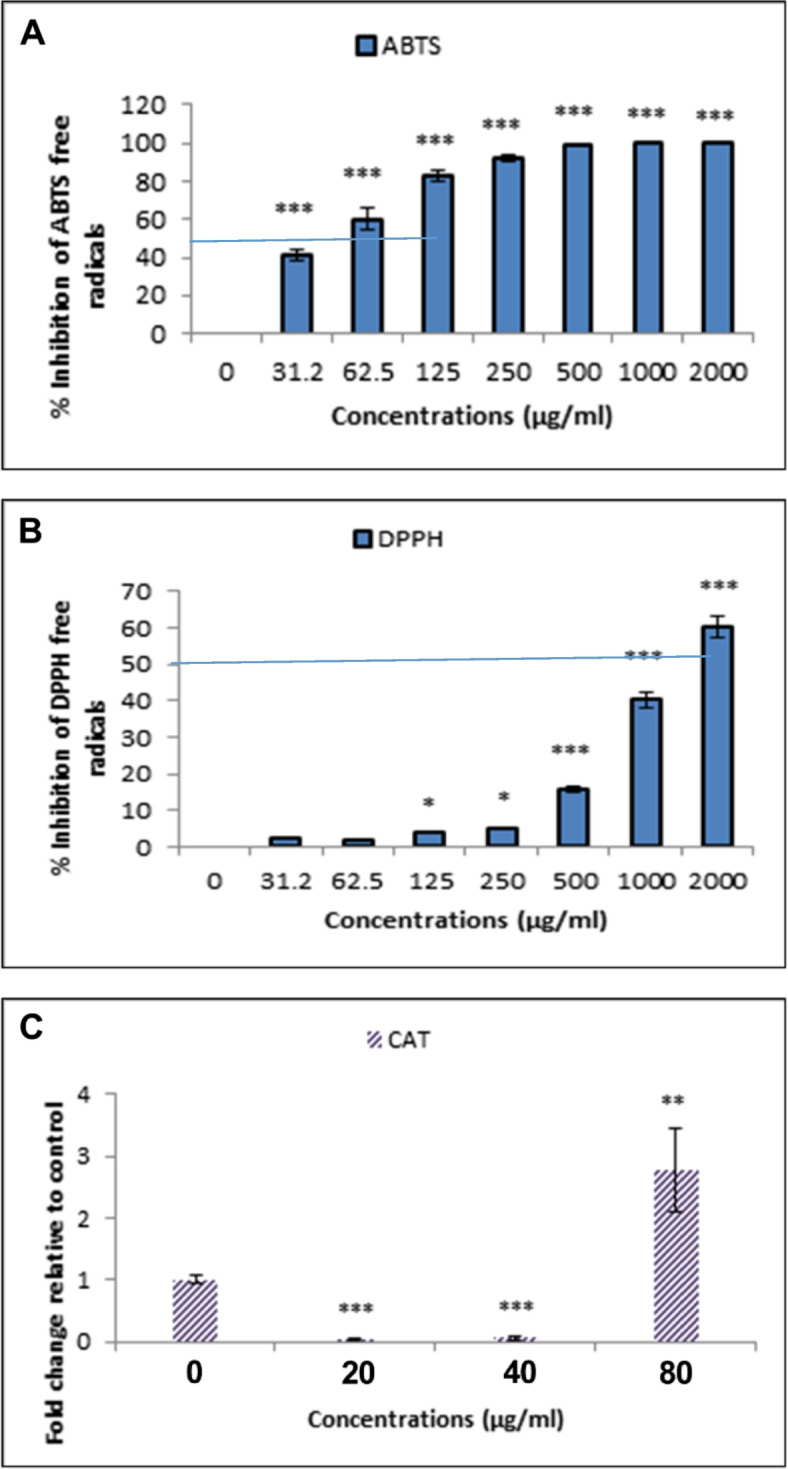
Antioxidant activity of MuSCF-NPs. **A** Effects of nanoparticles on ABTS; **B** Effects of nanoparticles on DPPH; **C** The Catalase gene expression. MuSCF-NPs: MuA-loaded SLN-Chotosan-folate nanoparticles

We investigated the inhibitory effect of nanoparticles on the ABTS free radicals. The minimum MuSCF-NP concentration scavenging the 50% of ABTS radicals was estimated at IC_50_ = 45 µg/mL. The ABTS inhibition rate met over 95% at greater than 250 µg/mL MuSCF-NPs concentrations. These results demonstrated a potent inhibitory effect of nanoparticles on ABTS free radicals in a concentration-dependent manner (Fig. 3A). The nanoparticles significantly inhibited the purple DPPH radicals in a concentration-dependent manner. The calculated IC_50_ value of MuSCF-NPs for DPPH suppression was reported at 1500 μg/mL (Fig. 3B).

#### Catalase gene expression

Due to the pivotal role of Catalase gene expression in regulating the cellular antioxidant defense system, its expression alteration determines the cell survival status [36]. As shown in Fig. 3C the significant decrease in CAT gene expression following the enhanced MuSCF-NPs concentrations (20 and 40 μg/mL) indicated the MuSCF-NPs’ good job in defeating the cancer cell antioxidant defense system. However, the overexpressed CAT gene in response the greater doses up to 80 μg/mL can be due to MuSCF-NPs’ potential to scavenge free radicals and give time to the cancer cell to activate its compensatory mechanisms and drug resistance strategies (Fig. 3C).

### Cytotoxic effect of nanoparticles on cancer and normal cells

**Fig. 4:**
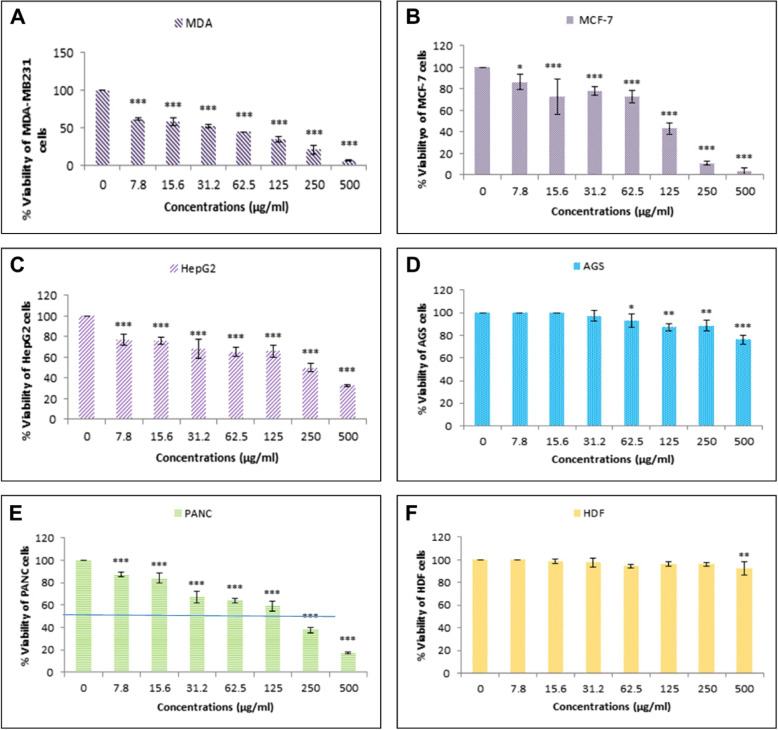
Cytotoxic effect of MuSCF-NPs on cancer cell lines compared to the normal cells. The viability of MDA-MB-231 (**A**), MCF-7 (**B**), HepG2 (**C**), AGS (**D**), PANC (**E**) cancer cells lines, and HDF (**F**) normal cell lines after treatment with various concentrations of nanoparticles. MuSCF-NPs: MuA-loaded SLN-Chotosan-folate nanoparticles

The produced nanoparticles significantly induced a selective cytotoxic impact on the human breast MDA-MB-231 cancer cells following their increased treatment doses compared with the other normal (HDF) and cancerous treated types including the AGS, PANC, HepG2, and MCF-7 cell lines in response at all concentrations. The IC_50_ concentration of MuSCF-NPs was determined at 40 μg/mL (Fig. 4A).

The 50%-decrease in MCF-7 breast cancer cells’ survival was detected at 11 μg/mL concentration of MuSCF-NPs (Fig. 4B). In this regard, the IC_50_ doses of MuSCF-NPs for the HepG2 liver, AGS gastric, and PANC pancreatic cancer cells were reported at 250 μg/mL, > 500 μg/mL, and 178.5 μg/mL concentrations, respectively (Fig. 4C, D, and E). The remarkable differences in cell survival results reflect the difference in cell sensitivity to the MuSCF-NPs, which can be concluded as the MuSCF-NPs safety and selectivity.

### Apoptotic effects of MuSCF-NPs

#### Flow cytometry and cell cycle status

**Fig. 5:**
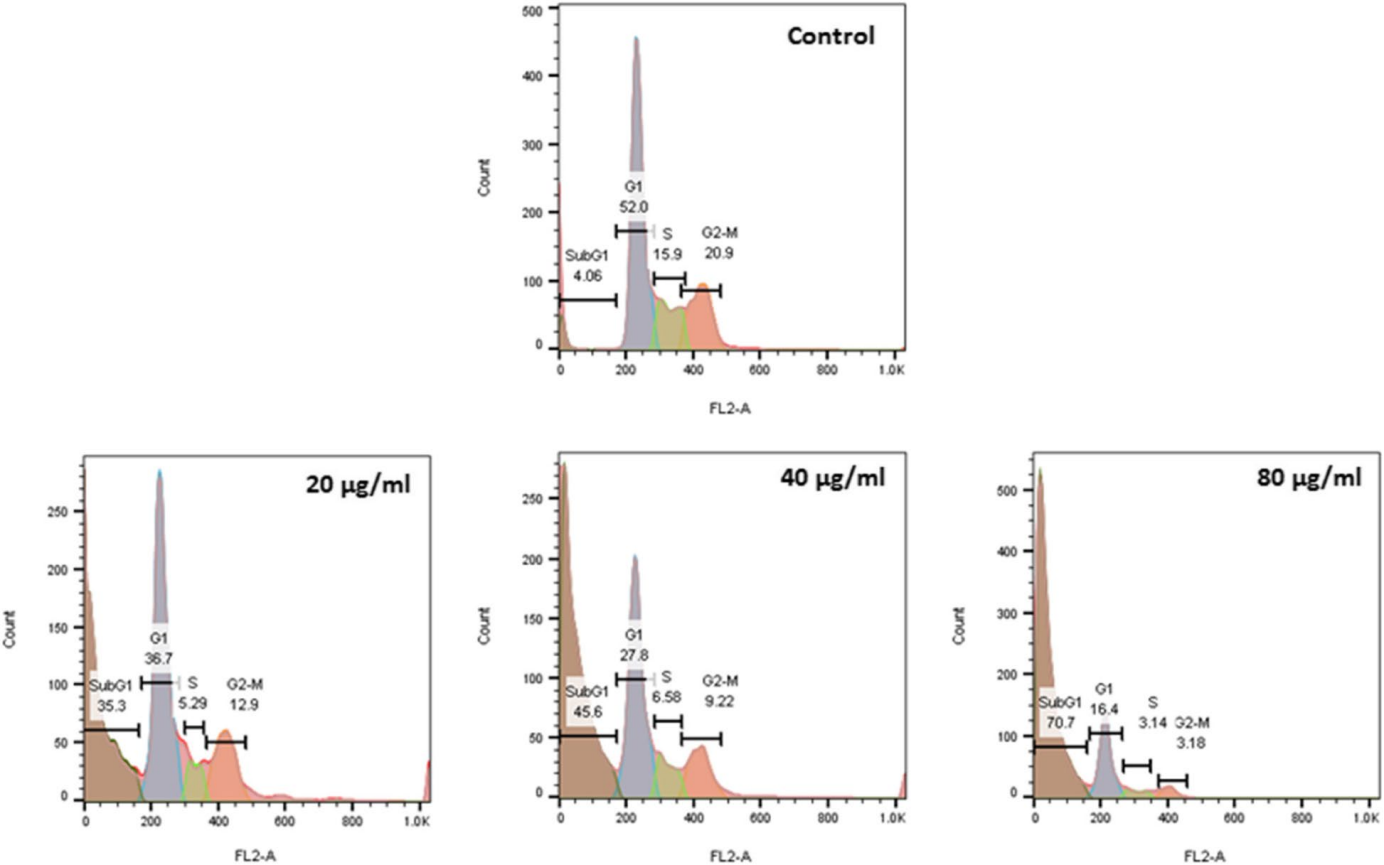
Anti-apoptotic effects of MuSCF-NPs. Effects of different concentrations of 20 µg/mL, 40 µg/mL, and 80 µg/mL of MUA-SCF-NPs on MDA-MB231 cells by flow cytometry method. MuSCF-NPs: MuA-loaded SLN-Chotosan-folate nanoparticles

The flow cytometry analysis exhibited a meaningful association between the enhanced SubG1-arrested cells and the increased Mu-SCF-NPs treatment doses. As shown in Fig. 4, the population of 4%-arrested cells in the SubG1 phase before the exposure process initiation has been increased to 35.3, 45.6, and 70.7% following the MuSCF-NPs’ exposure doses of 20, 40, and 80 µg/mL, respectively (Fig. 5). The increased arrested cells at the SubG1 phase of their cell cycle interval indicates the in-compensatable DNA injuries, which has led to activation of apoptotic pathways and suppression of cell cycle progression [37].

#### Expression of apoptosis genes

**Fig. 6:**
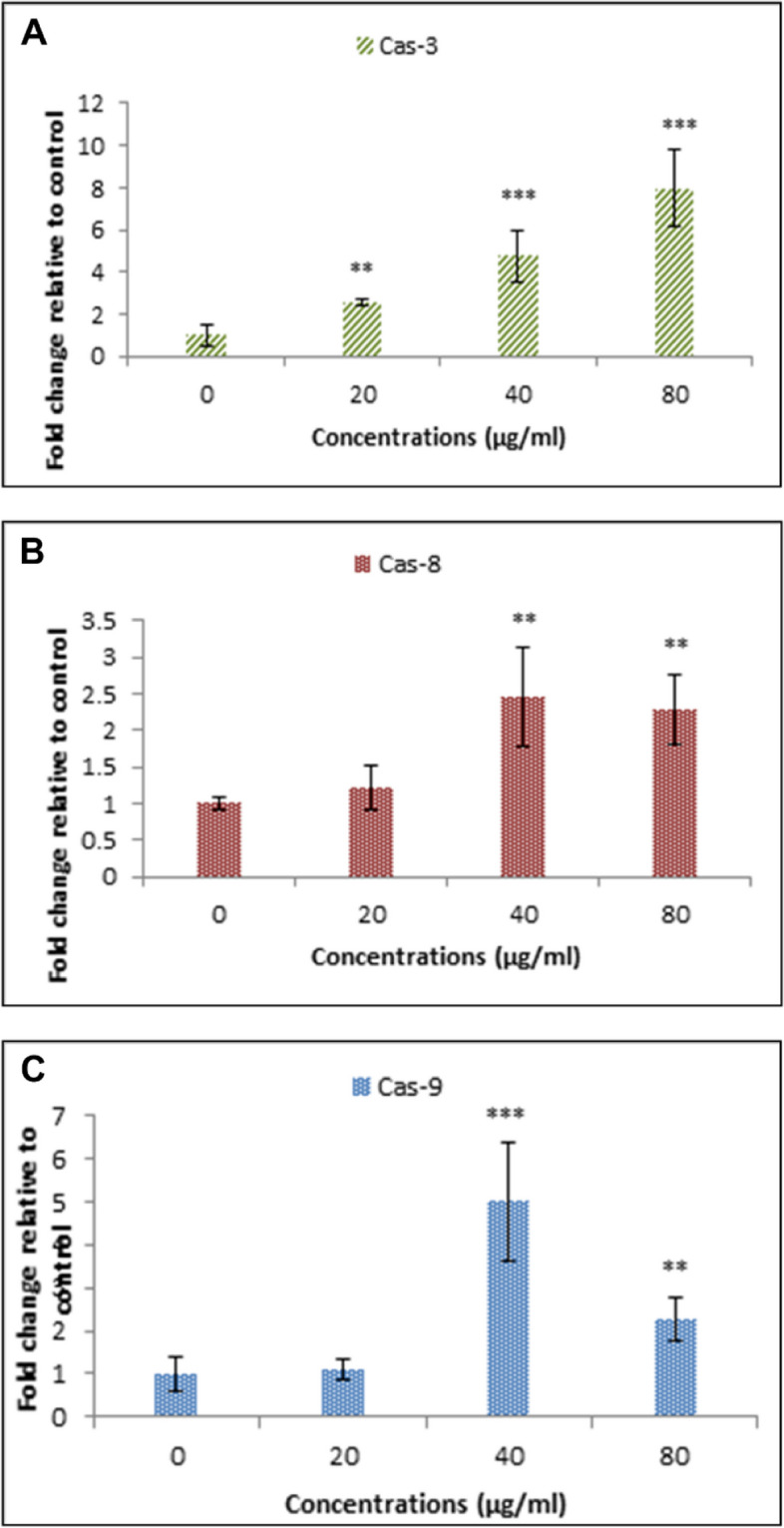
Apoptotic gene expression profile of MDA-MB231 breast cancer cells following the MuSCF-NPs treatment doses. The expression of Caspase-3 (**A**), Caspase-8 (**B**), and Caspase-9 (**C**) in MDA-MB-231 cells treated with different concentrations of nanoparticles. MuSCF-NPs: MuA-loaded SLN-chitosan-folate nanoparticles

In support of the cancer cell cycle status, the apoptotic gene expression profile of the MDA-MB-231 breast cancer cells exposed to different MuSCF-NPs concentrations was provided. The results indicated the notable meaningful overexpression of Caspase 3, 8, and 9 following the two first MuSCF-NPs doses (20 and 40 μg/mL) (*P*-value < 0.01) (Fig. 6). Therefore, both the external and internal apoptosis pathways have been activated by the MuSCF-NPs exposure, which refers to its powerful apoptotic activity on the human MDA-MB-231 breast cancer cells. The cell response to Caspase 8 and Caspase 9 expression pattern at higher doses of MuSCF-NPs, can be attributed to the cancer cell compensatory mechanisms and anti-apoptotic gene-regulators recovering process, which lead to the drug-resistant potential of cancer cells [38]. However, several biochemical pathways responsible for the cell drug resistance properties are involved.

## Discussion

The late diagnosis and poor prognosis of human breast cancer have made its treatment approximately impossible. However, there is hope for the development of functionalized nano-drug delivery systems as a potential substitute for chemotherapy. These systems offer the possibility of effectively suppressing cancer-related mortality [39]. To the best of our knowledge, this is the first study encapsulating MuA into the nano multi-protective delivery systems consisting of SLN matrix and decorated with folate-linked chitosan polymer to investigate its antioxidant and selective cytotoxic and apoptotic impacts on human breast cancer cells compared with other cancer types. The results revealed the powerful antioxidant and apoptotic activities of the produced MuSCF-NPs.

The design of functionalized nano drug delivery systems holds promise in revolutionizing cancer treatment. By utilizing nanoparticles as carriers for chemotherapy drugs, these systems offer several advantages. Firstly, they can enhance drug delivery to the cancer site and increase the concentration of the drug specifically within cancer cells while minimizing exposure to healthy tissues. This targeted approach can potentially improve treatment efficacy and reduce side effects. Additionally, functionalized nano-drug delivery systems can overcome biological barriers, such as poor solubility or rapid clearance, which often limit the effectiveness of conventional chemotherapy drugs [39].

There are several types of drug delivery systems based on their structural composition such as carbonic, polymeric, lipidic, metallic, etc. [40]. The SLN (Solid Lipid Nanoparticles) nano drug delivery systems as the lipidic based nanostructures have gained significant attention in the field of pharmaceutical research, which offer a promising approach for delivering phytochemicals bioactive compounds. Stearic acid is known for its high efficiency in drug entrapment. Also, the inclusion of ethanol and surfactant with an appropriate HLB (Hydrophilic-Lipophilic Balance) contributes to the homogeneity and colloidal stability of the formulation. By encapsulating phytochemical-loaded bioactive compounds within SLN, it becomes possible to enhance their solubility, stability and targeted delivery to specific sites in the human body. This approach holds great potential for improving the therapeutic efficacy and bioavailability of phytochemicals, opening up new possibilities for their utilization in the medical field [31, 41, 42].

Herein, the MuA as the natural cytotoxic and antioxidant compound was loaded into the SLN nanoparticles to be protected against oxidative reactions. In the current study, the formulated MuA antioxidant activity was verified by detecting both the increasing radical scavenging rate and Catalase gene down-regulation in the MDA-MB-NPs breast cancer cells, which led to a decrease in the exogenic ROS and increased the endogenic oxidative stress, respectively.

Catalase down-regulation in cancer cells reduced antioxidant capacity leading to ROS accumulation. Elevated ROS levels induce oxidative stress, DNA damage, lipid peroxidation, and protein oxidation [5, 36]. Therefore, designing a selective delivery mechanism for MuSCF-NPs plays an important role in preventing Catalase gene down-regulation in normal cells.

In this regard, the MuA-loaded SLN nanoparticles were coated with folate-linked chitosan nanoparticles to selectively target the cancer cells overexpressing the folate receptors such as breast, colon, lung, liver, ovarian, pancreas cancer cells [7–10]. The results showed a significant selective apoptotic impact on the human MDA-MB-231 breast cancer cells compared with other cell lines. The difference in cellular survival rate following the increased MuSCF-NPs concentrations reveals the role of folate receptor-mediated endocytosis mechanism as the cellular uptake process.

The interaction between the ligand and the receptor triggers the internalization process. The ligand-receptor complex is internalized through clathrin-coated pits, which invaginate and form vesicles known as clathrin-coated vesicles. These vesicles then undergo a series of intracellular trafficking events, including the shedding of the clathrin coat and fusion with early endosomes [32]. Within the early endosomes, the ligand can be either recycled back to the cell surface or transported to late endosomes and lysosomes for degradation. The folate receptor-mediated endocytosis pathway provides a selective and efficient means for delivering folate-conjugated therapeutics to cancer cells, exploiting the overexpression of folate receptors as a target for drug delivery [43, 44]. In this regard, the cells overexpressing folate receptors were more vulnerable to the MuSCF-NPs compared with those expressing a few numbers of folate receptors. Moreover, regarding the negative zeta charges of the cell membrane, the positively charged MuSCF-NPs can electrostatically interact with the target cell membrane, which improves the delivery mechanism. Therefore, based on the IC_50_ concentrations of MuSCF-NPs in normal cancer cells, the folate receptor expression density can be detectable in cancer cell lines.

On the other hand, the enhanced SubG1-arrested MDA-MB-231 cancer cell population following the MuSCF-NPs exposure refers to apoptotic death occurrence, which was verified by detecting the significant up-regulated Caspase 3, 8, and 9 gene expression.

Caspase 3, Caspase 8, and Caspase 9 are key players in the induction of both the intrinsic and extrinsic pathways of apoptosis. Caspase 3 is activated downstream of both pathways and plays a central role in dismantling cellular components during apoptosis. In the intrinsic pathway, Caspase 9 is activated in response to intracellular signals such as DNA damage or cellular stress. This activation occurs through the formation of a multiprotein complex known as the apoptosome, which leads to the cleavage and activation of Caspase 9. Once activated, Caspase 9 can then activate Caspase 3, initiating the execution phase of apoptosis. On the other hand, in the extrinsic pathway, Caspase 8 is activated by the binding of extracellular death ligands to death receptors on the cell surface. This receptor-ligand interaction triggers the assembly of a death-inducing signaling complex (DISC), leading to the activation of Caspase 8. Activated Caspase 8 can directly activate Caspase 3. Together, Caspase 3, Caspase 8, and Caspase 9 play critical roles in mediating apoptotic cell death through both intrinsic and extrinsic pathways, highlighting their importance as therapeutic targets for cancer treatment [45].

In the current study up to 40 µg dose of MuSCF-NPs up-regulated all the Caspases (3, 8, and 9), which indicates their potential in activating both apoptosis pathways.

Cytotoxic and pro-apoptotic effects of SLN-containing drugs or surface-modified nanoparticles have been reported in various studies [46–48]. The pro-apoptotic effect of SLN-containing Foeniculum vulgare plant extract on MCF-7 cancer cells was investigated [49]. In 2019, Liang et al. reported the pro-apoptotic effect of SLN nanoparticles by activating the internal pathways of apoptosis and disrupting the cell cycle. Their study showed that nanoparticles induced apoptosis in treated cells by causing an imbalance in Bax/Bcl-2 expression and increasing caspase-3 expression [50].

Herein, the SLN nanoparticles were modified by being loaded by MuA and functionalized by a folate-linked chitosan coating layer to manage the cellular uptake of MuA through the receptor-mediated endocytosis, which facilitates the selective nanocarriers’ arrival into the folate receptor positive cancer cells.

## Conclusion

SLN-containing drugs have a negative charge due to using some compounds such as lecithin and stearic acid. Furthermore, a negative charge on the surface of cancer cells can reduce the therapeutic properties of the synthesized nanocarrier. Therefore, using specific ligands with high expression in cancer cells can be a good part of effective strategies for drug delivery. This investigation showed a change in surface charge and a slight increase in the size of nanoparticles after modifying their surface with chitosan-folate, and the characterization of nanoparticles showed the synthesis of nanoparticles with suitable size, dispersion, and stability for clinical applications. Examining the amount of drug (Methyl urolithin A) encapsulation and folate binding also indicated the success of the nanoparticle preparation method. The results of this investigation confirmed the antioxidant, cytotoxicity, and pro-apoptotic effects of MuSCF-NPs, which makes the resulting nanoparticles suitable candidates for preclinical and clinical studies against breast cancer.

## Data Availability

The datasets generated during and/or analyzed during the current study are available from the corresponding author upon reasonable request.
